# Systematic review of indirect costs to families of children with developmental epileptic encephalopathies

**DOI:** 10.1186/s13023-025-04081-9

**Published:** 2025-11-12

**Authors:** Sunny Abdelmageed, Rebecca Y. Du, Maura Carroll, Anup Patel, Sandi Lam

**Affiliations:** 1 Division of Pediatric Neurosurgery, Ann and Robert H. Lurie Children’s Hospital, 225 E Chicago Ave, Box 28, Chicago, IL 60611 USA; 2 Department of Neurosurgery, Northwestern University Feinberg School of Medicine, Chicago, IL USA; 3 Division of Pediatric Neurology, Nationwide Children’s Hospital, Columbus, OH USA

**Keywords:** Caregiver, Developmental epileptic encephalopathies, Dravet syndrome, Indirect costs, Lennox-Gastaut Syndrome, Tuberous sclerosis complex

## Abstract

**Background:**

Severe childhood epileptic encephalopathies have high burden on families and large indirect healthcare costs. Several studies have examined indirect costs primarily in Tuberous Sclerosis and Dravet syndrome, finding impacts in all major aspects of life. Indirect costs of these diseases are reported to be higher than other severe pediatric illnesses. To better understand the epilepsy care journey and possible barriers to optimal medical and surgical care, we sought to understand indirect costs of care and burden of illness for families.

**Methods:**

A systematic search was conducted in accordance with the Preferred Reporting Items for Systematic Reviews and Meta-Analyses (PRISMA) guidelines using three databases (MEDLINE, Embase, and Scopus). Records were screened independently by two reviewers included based on pre-defined inclusion criteria. Studies were discussed narratively to identify common themes for analysis.

**Results:**

Of 2,084 publications, 24 studies met inclusion criteria. Indirect costs disproportionately impact mothers compared with fathers. Caregiver burden results in absenteeism and presenteeism. Many parents make major career changes, work part time, quit, or retire early due to caregiver responsibilities. Caregiving in this patient population is associated with decreased quality of life, primarily driven by increased depression, anxiety, stress, and poor sleep. Leisure time is sacrificed to fulfill responsibilities, which has negative impact on social connection and relationships, leading to feelings of isolation and reducing ability to cope. Siblings also face opportunity costs and psychosocial impacts. On a positive note, many parents report a sense of fulfillment and personal growth from caregiving roles.

**Conclusions:**

Indirect costs of pediatric developmental epileptic encephalopathies (DEE) are multifactorial. Caregiving for this patient population has negative economic, psychosocial, and physical impacts extending from the family unit to extended family and communities. Further studies are necessary to characterize and interventions that may improve the care journey, quality of life, and outcomes of patients and families living with DEE.

**Supplementary information:**

The online version contains supplementary material available at 10.1186/s13023-025-04081-9.

## Background

Families incur various indirect costs related to the burden of caring for a sick loved one in addition to direct medical expenses [1–3]. Indirect costs to caregivers are often reported by decreased work productivity, work loss, and worker replacement. Psychological, social, and physical impacts have also been described in caregivers [1, 4, 5]. For pediatric epilepsy, indirect costs to caretakers are enormous [6–9]. Pediatric epilepsy is the most common chronic neurological disorder in children, and the indirect costs are estimated to contribute up to 85% of total annual costs for the care of children with epilepsy [8]. With total costs estimated at up to $11,432 in high-income countries in 2022 [10]. Over half of caregivers of children with epilepsy have psychopathological symptoms, including high levels of stress leading to post-traumatic stress disorder, depression, anxiety, and sleep disturbances [5, 11–13].

The term developmental and epileptic encephalopathies (DEE) was developed in 2017 to describe conditions in which both developmental impairment and epileptic activity contribute negatively to the cognition and behavior of the patient [14]. Lennox-Gastaut Syndrome (LGS), Dravet Syndrome (DS) and Tuberous Sclerosis Complex (TSC) are severe forms of childhood onset DEE. Seizures often begin in the first few years of life, and almost all of these children will have developmental and intellectual delay, leading to decreased health-related quality of life (QOL) and major caregiver burden [15–17].

Understanding and quantifying indirect costs are essential for decision-making, healthcare resource allocation, and in defining the support required by families to improve health outcomes [2, 18]. Several studies have examined the indirect healthcare costs associated with DEE, primarily focusing on populations with DS and TSC [19–21] identifying costs in all aspects of life [22–24]. These studies use a variety of methods to explore various indirect costs and often focus on one or two domains such as physical health or psychosocial effects. While these findings are helpful, the burden of these conditions is multifaceted, and comparing caregiver burdens across different DEEs could provide additional insights.

We conducted a systematic review of the current literature on indirect costs of DEE, specifically, LGS, DS, and TSC, to delineate the secondary consequences of caregiver responsibilities and unquantified burdens.

## Methods

A systematic review was performed according to the Preferred Reviews and Meta-Analyses (PRISMA) 2020 guidelines to explore the indirect costs of childhood epilepsy syndromes [25]. PubMed MEDLINE (National Library of Medicine), Embase (Elsevier), and Scopus (Elsevier) were searched on May 10, 2023 using keywords associated with three childhood epilepsy syndromes (Lennox-Gastaut Syndrome, Dravet Syndrome, Tuberous Sclerosis), pediatric populations, and indirect healthcare costs. A supplemental table shows this in more detail (see Supplemental File 1 for a full list of search terms). Articles were restricted to English language. No article type restrictions were applied. All studies meeting inclusion criteria were included from inception of the databases to May 10, 2023.This protocol was not prospectively registered because screening and data extraction were initially completed for a grant submission.

After the initial search, duplicates were excluded, and the remaining articles were screened for relevance by title and abstract based on the following prespecified inclusion criteria: 1) published in or translated into the English language, 2) available full text, 3) population of patients with LGS, DS, TSC or another severe childhood-onset epilepsy syndromes, and 4) providing outcomes of indirect healthcare costs. Articles progressing to full-text review were screened for final inclusion based using the full prespecified inclusion criteria seen in supplemental Table 2. Indirect health care costs included indirect economic cost (e.g., productivity loss), psychosocial impacts, physical impact, and impact on siblings. Multi-pass deduplication was performed using EndNote (Clarivate Analytics, Philadelphia, PA) and eligible articles were screened using Rayyan (https://rayyan.qcri.org/). This systematic review was conducted independently by two reviewers and disagreements were resolved based upon discussion.

Data were extracted independently by authors and cross-checked for accuracy. Included articles were reviewed for bibliographic data, design, participants, and outcomes. For each study, the population, country of origin, study design, and indirect costs were recorded. In addition, qualitative themes identified through qualitative studies were recorded.

Critical appraisal of included studies included risk of bias assessment using the Risk of Bias in Non-randomized Studies-of Interventions (ROBINS-I) tool conducted by adapting study design grades from Shadish et al. [26, 27]. Two reviewers (SA and RD) independently assessed each study. A third reviewer resolved disagreements through consensus.

## Results

**Fig. 1:**
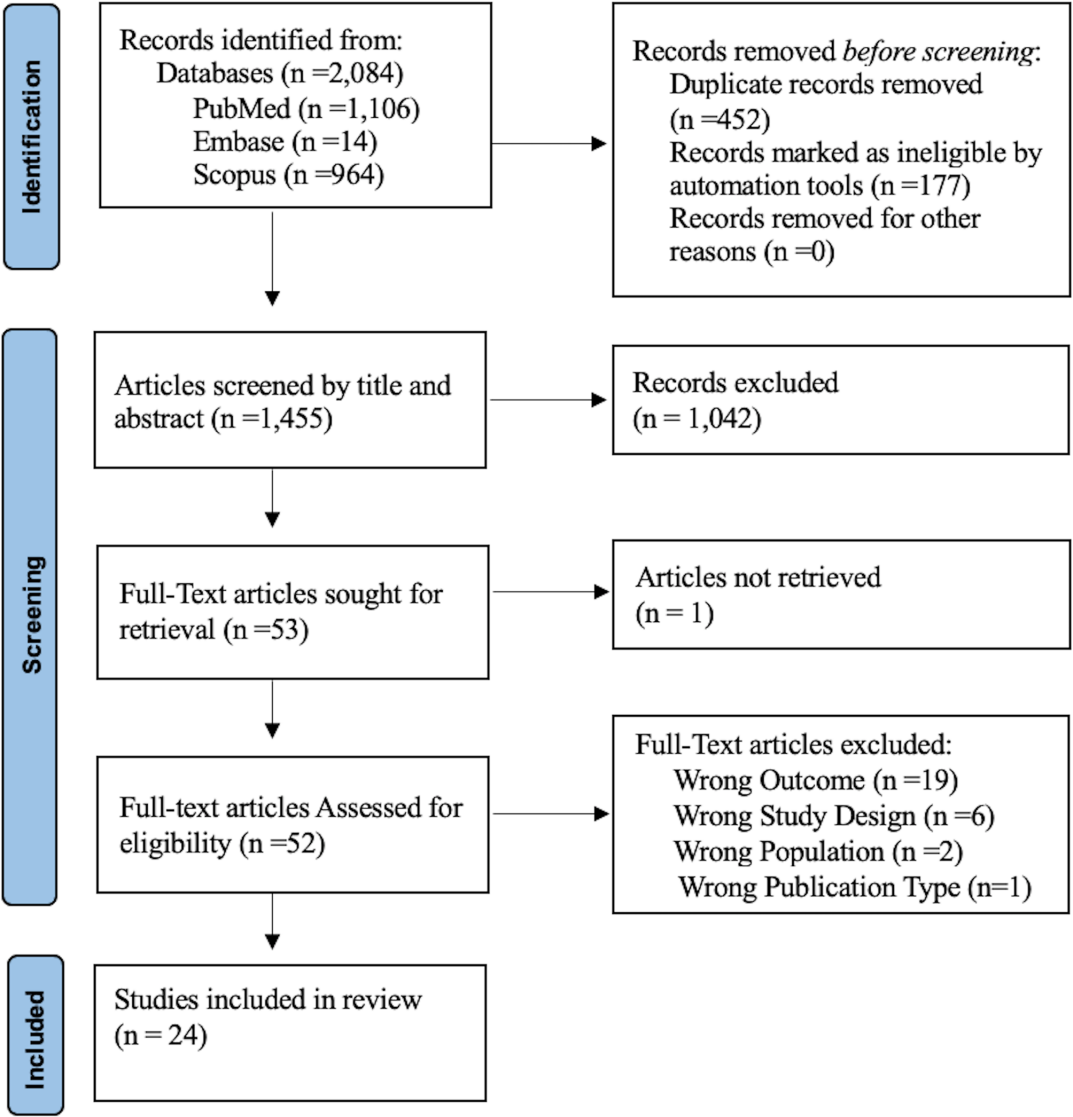
PRISMA flowsheet of included full-text articles

From the three databases, 2,084 records were identified. Following de-duplication 452 duplicates were removed and 177 were marked as ineligible by automation tools, due to non-English language or animal studies, these were cross-checked for accuracy by author SA. A total of 1,455 abstracts were screened and 52 were included for full text review. After screening using pre-determined inclusion/exclusion criteria, 24 full text articles were included in this review (see Fig. 1 for PRISMA full text selection flowchart). The overall quality of evidence was grade D (All studies were either cross-sectional studies or semi-structured interview) as per grading recommendations described in Shadish et al. The overall risk of bias was high.

**Fig. 2:**
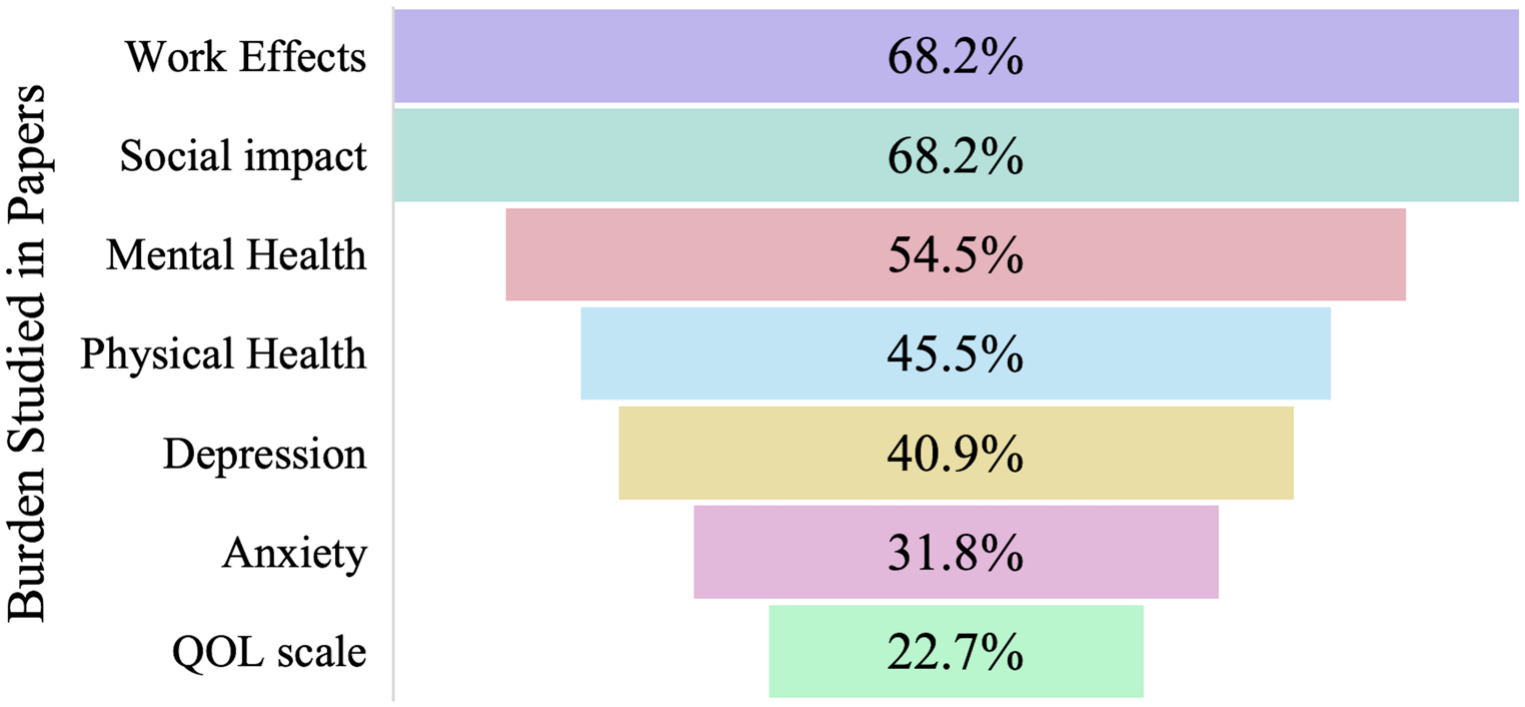
Burden studied in papers. Funnel plot demonstrating percent of manuscripts that reported each category of burden. QOL, quality of life

Study characteristics are shown in Tables 1 and 2. Indirect costs for DS caregivers were reviewed in 54.2 of manuscripts (*n* = 16), 33.3% for TSC (*n* = 8), 16.7% for LGS (*n* = 4), and 12.5% included other unspecified childhood onset epilepsies in addition to LGS, Dravet, or TSC (*n* = 3). Numbers do not add up to 24 as some papers combined diagnoses in the study population. Qualitative methods were used in 29.2% (*n* = 7) of studies, 66.68 (*n* = 16) used quantitative methods, and 4.1% (*n* = 1) used both. Qualitative methods consisted of semi-structured interviews or focus groups. All quantitative studies were cross-sectional designs. Results were sorted into themes based on primary outcomes. The psychosocial theme comprised 79.2% of manuscripts (*n* = 19), 62.5% in “economic” (*n* = 15), 41.7% in “physical” (*n* = 10), and 12.5% 3 in “siblings” (*n* = 3). Themes were further broken down into work effects (68.2%), social impact (68.2%), mental health effects (54.5%), physical effects (45.5%), and quantitative QOL impacts (22.7%, Fig. 2). Within mental health, 40.9% of papers described depression and 31.8% described anxiety. Studies that did include demographic and socioeconomic data showed subjects were primarily female (56–93%), highly educated (college degree or higher 66–79%), and White/Caucasian (84–91%). Fathers were more likely to be employed than mothers 82–91% compared with 46–70%.

**Table 1:** Summary of included articles

Author, Year	Population	Country	Methods		Themes
Bailey, 2020 [28]	LGS, Dravet, TSC, other	United States	Quantitative	Cross-sectional multicenter survey	Sibling
Campbell, 2018 [29]	Dravet	United States	Quantitative	Cross-sectional survey single-center survey	Economic Psychosocial
Domaradzki, 2023 [30]	Dravet	Poland	Quantitative, qualitative	Cross-sectional multicenter survey	Economic Psychosocial
Gallop, 2010 [19]	LGS	United Kingdom	Qualitative	Semi-structured interviews	Economic psychosocial physical
Gibson, 2014 [31]	LGS	United States	Qualitative	Cross-sectional open and close-ended survey	Psychosocial
Graffigna, 2013 [32]	TSC	Italy	Qualitative	Semi-structured interviews	Psychosocial physical
Grau, 2021 [20]	TSC	Germany	Quantitative	Cross-sectional multicenter survey	Economic
Hesdorffer, 2020 [33]	Other	United States	Quantitative	Cross-sectional design using REN data	Psychosocial physical
Jansen, 2020 [34]	TSC	31 countries	Quantitative	Cross-sectional multicenter survey	Psychosocial
Jensen, 2017 [35]	LGS, Other	United States	Qualitative	Focus groups	Psychosocial physical
Kopp, 2008 [24]	TSC	United States	Quantitative	Cross-sectional single-center survey	Psychosocial
Lagae, 2019 [36]	Dravet	UK, France, Germany, Spain, Italy	Quantitative	Cross-sectional multicenter survey	Economic Sibling
Maltseva, 2023 [37]	Dravet	Germany	Quantitative	Cross-sectional survey, prospective diary	Economic psychosocial physical
Nabbout, 2018 [38]	Dravet	France	Qualitative	Semi-structured interviews	Economic psychosocial physical
Nabbout, 2019 [39]	Dravet	United States, United Kingdom, Italy	Qualitative	Semi-structured interviews	Economic psychosocial physical
Nabbout, 2020 [40]	Dravet	France	Quantitative	Cross-sectional multicenter survey	Economic psychosocial physical
Gil-Nagel, 2023 [41]	Dravet	Spain	Quantitative	Cross-sectional multicenter survey	Economic, psychosocial physical
Nolan, 2006 [42]	Dravet	Canada	Qualitative	Semi-structured interviews	Psychosocial sibling
Rentz, 2015 [43]	TSC	United States	Quantitative	Cross-sectional survey	Psychosocial physical
Skalicky, 2018 [44]	TSC	United States	Quantitative	Cross-sectional survey	Economic
Strzelczyk, 2019a [45]	Dravet	Germany	Quantitative	Cross-sectional, multi-center survey, prospective work productivity diary	Economic, psychosocial
Stzrelczyk, 2019b [46]	Dravet	Germany	Quantitative	Cross-sectional, multicenter survey,	Economic psychosocial
Whittington, 2017 [47]	Dravet	United States	Quantitative	Cross-sectional survey	Economic
Willems, 2021 [21]	TSC	Germany	Quantitative	Cross-sectional multicenter survey	Economic Psychosocial

LGS, Lennox-Gastaut Syndrome; TSC, tuberous sclerosis complex

**Table 2:** Study characteristics

	Aim of study	Mean Age or Range	Demographics of Caregivers	Number of Caregivers	Outcomes	Instruments Used
Bailey, 2020 [28]	Impact on siblings of DEE	Patients: 16 Siblings: 21 Caregivers: NR	NR	128 Siblings: 120	QOL Psychosocial impact Burden	Sibling Voices Survey
Campbell, 2018 [29]	Impact on caregivers of DS	Patients: 11.7 Caregivers: NR	NR	30	Caregiver burden QOL Economic burden	OCBS EQ-5D-5 L General Health Assessment WPAI NHIS
Domaradzki, 2023 [30]	Emotional experience of DS caregivers	Caregivers: 39.7	90.7% female 22.7% employed full-time	75	Emotional experience and burden of caregiving QOL	Standardized questionnaire
Gallop, 2010 [19]	QOL for caregivers and LGS children	Patients: 12* Caregivers: 39*	90% female	40	QOL Psychosocial impact Health status	SF-36 HADS
Gibson, 2014 [31]	Impact of LGS	Patients: 3.5–36 Caregivers: NR	NR	96	Psychosocial impact Financial impact Impact on family	Survey
Graffigna, 2013 [32]	Parent experience with TSC	Patients: 12 Caregivers: 47	64.6% female 100% Italian	48	Caregiver experience	Qualitative interview
Grau, 2021 [20]	Direct and indirect costs of TSC	Patients: 9.8 Mothers: 40,8 Fathers: 43.4	Mothers: 70% employed Fathers: 91% employed	184	Disease severity Healthcare utilization Direct and indirect costs	Questionnaire
Hesdorffer, 2020 [33]	Caregiver sleep in DEE	Caregivers: 40	91.9% female 91% NHW 60.2% employed (full- or part-time)	742	Disease severity Fatigue Sleep disturbance QOL	REN survey PROMIS
Jansen, 2020 [34]	Burden of illness and QOL in TSC	Patients: 19.8 Caregivers: NR	61.5% female	143	Burden of illness Healthcare utilization QOL	EQ-5D QOLCE
Jensen, 2017 [35]	Life impact for DEE caregivers	Patients: 8.4 Caregivers: 42	84% female 84% NHW 26% employed full-time	19	Physical health Mental health Social function Financial resources	PROMIS
Kopp, 2008 [24]	Behavior Problems in TSC	Patients: 7.7 Caregivers: NR	75.6% female 66.7% employed	45 Patients: 99	Psychological impact Parental stress	Parenting Stress Index SCL-90-R
Lagae, 2019 [36]	Caregiver impact and healthcare utilization in DS	Caregivers: NR	NR	584	QOL Support Out-of-pocket expenses Treatment	Online Survey EQ-5D-5 L
Maltseva, 2023 [37]	Sleep and caregiver burden in DS caregivers	Patients: 13.5 Caregivers: 44.7	Primary caregivers: 92.6% female Mothers: 46.3% employed Fathers: 85.2% employed	108	Psychosocial impact Physical health Caregiver burden Professional impact	HADS PSQI BSFC Questionnaire Prospective diary
Nabbout, 2018 [38]	Caregiver related outcomes in DS	Caregivers: 40.7	57.1% female	11	Psychosocial impact Physical Caregiver burden QOL	Qualitative interview
Nabbout, 2019 [39]	Impact of DS in families	Caregivers: 43.3	65% female	20	Caregiver burden Physical health Psychosocial impact Family impact	Qualitative interviews
Nabbout, 2020 [40]	Impact of DS on caregivers	Patients: 7.6* Caregivers: NR	79.3% college education or greater	87	Caregiver burden Psychosocial impact Professional impact	Survey
Gil-Nagel, 2023 [41]	Impact of DS on QOL and Family	Patients: 10.8 Caregivers: NR	NR	80	QOL Professional impact	Carer-QOL SINDRA HUI
Nolan, 2006 [42]	Coping in DS	Patients: 10.2 Caregivers: NR	NR	24	Coping ability Healthcare utilization	ICND
Rentz, 2015 [43]	Caregiver burden in TSC	Patients: 6.9 Caregivers: 39	78.9% female 86% NHW 65.5% college education or greater 63% employed full-time	275	Psychosocial impact QOL Physical health Healthcare utilization	Survey SF-12 HDI
Skalicky, 2018 [44]	Cost of illness in TSC	Caregivers: 39	79% female 86% NHW 66% college education or greater 64% employed	275	Healthcare utilization Economic burden	Survey WPAI
Strzelczyk, 2019a [45]	Cost and Burden of DS	Patients: 10.1 Mothers: 42.1 Fathers: 45.2	Mothers: 56% employed Fathers: 82% employed	93	Psychosocial impact QOL Economic burden Healthcare utilization	EQ-5D-3 L BDI-II
Stzrelczyk, 2019b [46]	Cost and burden of DS	Patients: 8.1	62% employed	93	Psychosocial impact QOL Economic burden Healthcare utilization	EQ-5D-3 L BDI-II
Whittington, 2017 [47]	Direct and indirect costs of DS	Caregivers: NR	72.73% employed	34	Direct and indirect costs Economic burden	NHIS WPAI
Willems, 2021 [21]	QOL in TSC and caregivers	Patients: 9.8 Caregivers: 41	84.8% female	184	Psychosocial impact QOL Professional impact	EQ-5D BDI-II

*Median value reported

Abbreviations: DEE, developmental epileptic encephalopathy; QOL, quality of life; DS, Dravet Syndrome; OCBS, Oberst Caregiving Burden Scale; EQ-5D-5 L, 5-level EuroQol-5 Dimensions; WPAI, Work Productivity and Activity Impairment Questionnaire; NHIS, National Health Interview Survey; LGS, Lennox-Gastaut Syndrome; SF-36, 36-Item Short Form Health Survey Questionnaire; HADS, Hospital Anxiety and Depression Scale; TSC, Tuberous Sclerosis Complex; NHW, non-Hispanic White; REN, Rare Epilepsy Network; PROMIS, Patient-Reported Outcomes Measurement Information System; QOLCE, Quality of Life of Childhood Epilepsy Questionnaire; SCL-90-R, Symptom Checklist-90-Revised; PSQI, Pittsburg Sleep Quality Index; BSFC, Burden Scale for Family Caregivers; SINDRA, survey used to assess patient health-related quality of life; HUI, Health Utility Index; ICND, Impact of Childhood Neurological Disability Scale; SF-12, 12-Item Short Form Health Survey Questionnaire; HDI, Hamilton Depression Inventory; BDI-II, Beck’s Depression Inventory Second Edition

### Indirect economic costs

The indirect economic costs as measured by impact on work are summarized in Table 3. Fifteen studies evaluated the economic impact and productivity loss due to the burden of caregiving [19–21, 29, 30, 32, 36–41, 44–47]. Primary outcomes were absenteeism and presenteeism. Mothers were significantly more impacted by productivity loss than fathers. Caregiving was primarily provided by unpaid caretakers.

**Table 3:** Indirect costs of caregiving on work, quality of life, mental health, and opportunity cost

Study	Reported Indirect Costs
Campbell, 2018 [29]	• 78% unable to work, 45% quit/lost job, 18% altered work situation • 70% slight anxiety, 33% moderate anxiety, 57% physical discomfort/pain, decreased QOL, • 27 fewer leisure hours
Domaradzki, 2023 [30]	• 77.3% unable to work • 80% mental exhaustion, 81.3% physical fatigue, 74.7% negatively impacted QOL, 46.7% impacted sleep, • 66.6% leisure time given up, 46.7% negative impact on relationships, 73.4% lack of time for personal development, 41.4% feel isolated
Gallop, 2010 [19]	• 57.6% moderate-severe anxiety, 51.5% depression, 24.2% moderate-severe, decreased mental health component score compared to US GP • Decreased social functioning compared to GP
Gibson, 2014 [31]	• 55% negatively impact activities, 13% brought partner further, 40% no time for other children
Graffigna, 2013 [32]	• 15% request emotional counseling • 50% unmet caregiver needs, 25% request more psychosocial support
Grau, 2021 [20]	• 13% (mothers), 1.1% (fathers) quit/lost job, 60.3% altered work situation
Jansen, 2020 [34]	• 58.8% unable to work, 21.2% quit/lost job, 34% altered work situation, 53.2% transitioned to part-time • 58.8% anxiety, 45.6% moderate-extreme, 70.7% depression, 53% pain/discomfort • Negative impact on relationships, 40.8% family, 50.7% social, 23.9% work colleagues
Jensen, 2017 [35]	• Increased anxiety, depression, sleep disturbance, PROMIS scores compared to GP, decreased physical conditioning compared to GP. • Decreased social functioning compared with GP
Kopp, 2008 [24]	• 12.9% significant anxiety, 56.3% significant depression, 46.5% significant impacted QOL
Lagae, 2019 [36]	• 81% of unemployed gave up job for caregiving, 65% took time off in last 4 weeks • 77% < 1 hr of leisure time/day, 46% of siblings gave up leisure time, 70% negative impact on family relationship, 80% impact on social relationships
Maltseva, 2023 [37]	• 33.3% (mother), 0.9% (father) Quit/lost job, 70.4% (mothers), 5.6% (fathers) altered work situation, 26.2% (mothers), 2.8% (fathers) reduced working hours • 35.3% significant anxiety, 23.1% significant depression, increased anxiety/depression HADS score compared to GP, 76.9% sleep disturbance
Nabbout, 2018 [38]	• 67% unable to work • 100% sleep disturbance • 100% no free time, 67% impact on leisure, negative impact on relationships 67% spouse, 78% family, 100% social
Nabbout, 2019 [39]	• 90% negative impact on work trajectory • 60% negative impact on emotional wellbeing, 70% physical impact, 75% impacted sleep • 80% impacted leisure time, 60% impact on social life, 90% negative impact on family relationships
Nabbout, 2020 [40]	• 33% of mothers unable to work, 58% (mothers), 40% (fathers) altered work situation, 52% (mothers), 7.1% (fathers) interrupted work > 6 m • 19.5% mothers, 15.3% fathers rated health poor/very poor • > 50% negative impact on relationships
Gil-Nagel, 2023 [41]	• 48.1% quit/lost job, 65.8% took time off • Mental health problems, 46.3% (some), 22.5% (a lot), 61.3% physical health problems • 35% negative impact on relationships
Nolan, 2006 [42]	• Negative impact on relationships, 45.8% spouse, 37.5% family, 62.5% friends, 70% difficulty finding caregiver
Rentz, 2015 [43]	• Decreased social functioning compared with GP • 42% mild to severe depression, decreased physical and mental component scores compared to GP
Skalicky, 2018 [44]	• 11% missed work, 38% impaired work time
Srtzelczyk, 2019a [45]	• 31% (mother), 1% (father) Quit/lost job, 29% (mothers), 6% (fathers) reduced working hours • 38.2% anxiety, 46% depression, decreased QOL scores on EQ-VAS compared to GP
Strzelczyk, 2019b [46]	• 28% quit/lost job, 29% reduced working hours • Increased BDI scores, decreased QOL scores on EQ-VAS compared to DRE and SR groups
Whittington, 2017 [47]	• 27.27% quit, 18.18% lost job, 18.18% altered working situation • 100% lost leisure time 2047 hrs/yr
Willems, 2021 [21]	• 13% (mother), 1.1% (father) Quit/lost job, 5.4% (mothers, fathers) altered work situation, 84.8% (mothers), 10.3% (fathers) reduced working hours • 45.7% depression, decreased EQ-VAS

Significant indicates a score on the utilized scale that equates to a clinically significant level of pathology and/or statistically higher value than the general population

Nine papers reported 2.8–84.8% of caregivers took meaningful amount of time off work or reduced their working hours due to caregiving responsibilities [21, 34, 36, 37, 40, 41, 44–46]. This was reflected more among mothers than fathers, with estimated percentages ranging between 26.2–84.2% vs 2.8–29% respectively. Grau et al. and Strzelcyk et al. quantified the indirect productivity costs using the human capital method in two German cohorts and found them to be to be as high as €1109 for mothers and €163 for fathers (see Table 4) [20, 45]. This discrepancy is attributed to the significantly greater reduction in work hours among mothers compared to fathers. Grau et al. looking at a TSC cohort, report a higher economic burden than Strzelczyk et al. who assessed a Dravet syndrome cohort. This may suggest that TSC causes a greater indirect economic burden; however, more studies are needed [20, 45]. For the entire Dravet syndrome cohort, total productivity costs over 3 months were €4,398.6 for mothers and €358.2 for fathers, which were greater compared with the drug-resistant epilepsy (DRE) cohort (€1,469 for mothers and €72 for fathers) [46].

**Table 4:** Quantified indirect economic cost

		Reduced hours (€/3 m)	Missed work (€/3 m)	Productivity (€/3 m)
Grau, 2021 [20]	Mothers	€1109	€732	€1466
	Fathers	€86	€163	€122
Srtzelczyk, 2019a [45]	Mothers	€238	€496	
	Fathers	€155	€127	
Whittington, 2017 [47]			$2500	$3000

(€/3 m): Euros over a 3-month period, (yr): year

Whittington et al. found employed caretakers reported missing an average of 47.6 eight-hour workdays (nearly 20% of total work hours) annually due to caregiving responsibilities, resulting in annual loss of ~$7,500 ($2500/3 m) per patient with DS [47]. This is significantly higher than the economic cost reported by both Grau et al. and Strzelczyk et al.; however, this study examines a U.S. cohort compared to a German cohort, and this difference likely reflects variations in caregiver salaries between these two countries. Lagae et al. estimated annual out of pocket fees for childcare paid by caregivers to be $3371 (135–40,449) [36]. While Srtzelczyk et al. estimated informal care provided by family members to be valued at €1130 over 3 months [46].

Up to two-thirds of caregivers changed jobs or lost their employment due to caregiving responsibilities. Eight papers report 5.4–90% of caregivers had to alter their work situation due to caregiving [20, 21, 29, 34, 37, 39, 40, 47]. Whittington et al. found an average salary reduction of $16,053 [47]. Eleven papers reported 0.9%-48.1% of caregivers either quit or lost their job, and Lagae et.al found that 81% of already unemployed caregivers (34% of the study demographic) reported leaving their prior employment due to caregiving responsibilities [20, 21, 29, 34, 36, 37, 40, 41, 45–47]. Mean productivity costs over a three-month period were estimated at €1466, for mothers who quit working (€122 for fathers) [20]. Five papers demonstrated 33–38% of caregivers reported an inability to work due to caregiving responsibilities [29, 30, 34, 38, 40]. Selected statements made by caregivers are summarized in Table 5.

**Table 5:** Caregiver statements

Study	
**Indirect economic cost**	
Nabbout, 2018 [38]	“My wife can’t work today, the attention on X is too important, and to leave her with somebody else is impossible today. I just have my license as a nurse and afterwards we discovered X’s illness and I saw my dream go away”
Gallop, 2010 [19]	“I was teaching in a secondary school. I only did six weeks because you just can’t cope with … so I gave up the teaching, and we’re in the, sort of, if you like, problem, not you know, we’re not absolutely desperate for money, but we’re very worried”
Quality of life, depression, anxiety, and stress	
Graffigna, 2013 [32]	“It is the disease of anguish: you always live in a state of anxiety about the future”
**Impact on physical health**	
Jensen, 2017 [35]	“I actually think one of the biggest differences in caring for a child with epilepsy versus maybe other chronic illnesses is your uncertainty at night, and the profound lack of sleep … The idea that you never punch out … is hard … I think you’re already dealing with something so emotionally heavy and physically exhausting during the waking hours that the sleep component is just a crazy facet of caregiving for someone with epilepsy”
**Opportunity cost of caregiving responsibilities**	
Nabbout, 2018 [38]	“Emotionally we have a hard time getting involved in anything. How can you work when you know that the center of your life is your child’s condition. We are kind of detached from the day-to-day life of others”
Jensen, 2017 [35]	“We don’t even make plans to do things with him because with him being home, keeping the house a little darker, a little quieter, keeps us from spending any time in the hospital, such is the life we choose … I’ve accepted it. It’s what it is. Whatever gets you through the day, that’s what we do”
Gallop, 2010 [19]	“It’s very difficult to plan anything or do anything because you really didn’t know, you know, if people invited you out you didn’t whether you were going or not going right up until the last minute, because, you know, you could be in hospital before you knew where you were with him. You just didn’t know. You couldn’t plan anything”
**Indirect costs are a reinforcing cycle**	
Jensen, 2017 [35]	“We gave up everything. We gave up our house … Just all that. I mean, we gave up all kinds of stuff. I mean, all the things we had worked up to. All the things we had purchased. All the things that we ever owned: A camper, a boat. We had to give them up too also because for him to qualify for Social Security and to get the things that he needed, you have to be poor to get those things”
Nabbout, 2018 [38]	“She has trouble speaking, she doesn’t talk like you and me, she has trouble articulating and she is constantly finding her words. She has a restricting vocabulary …she doesn’t speak correctly, and we have to figure out what she is trying to say”
**Positive impact of caregiving responsibilities**	
Jensen, 2017 [35]	“I have full confidence in myself in what I’m doing with him. I have no doubts of what I’m doing. I mean, just because of what we’ve seen him through and just—you know, we now know after so many hospital visits that, you know, when you go in, you have to be his advocate. You have to ask questions. You have to demand things. You have to tell people; this is how I want it done. You know, because it’s so unique as far as in a hospital stay or just a group stay, or whatever. They don’t know, most people don’t know how to keep a kid with Dravet, how to keep them safe and all that kind of stuff. So it’s boosted my confidence with both [my son] and his sister as far as parenting”

Several studies also commonly found presenteeism among caretakers. Whittington et al. found employed caretakers reported ~77 workdays of annual presenteeism, resulting in approximately $12,000 per patient annually [47]. Caregivers reported 38% of work time was impaired due to TSC caregiving [44]. Jansen et al. found that 36.2% of caregivers reported that TSC caregiving negatively impacted their career progression [34].

Additional indirect economic costs arise from caregivers rearranging their families’ living situations to accommodate patient needs. Nabbout et al. found nearly 20% of families moved to a new house to be closer to their hospital or because they required a home better adapted to their child’s needs [38, 40].

### Quality of life, depression, anxiety, and stress

The reported impacts on quality of life, depression, anxiety, stress and physical health in caregivers are summarized in Table 3. Caregivers have higher levels of anxiety and depression compared to the general population. Seven papers reported anxiety in as high as 70% of caregivers [19, 24, 29, 34, 35, 37, 45]. Nine papers reported depression in caregivers with ~23.3% reporting moderate-severe depression and as high as 70.7% with some depression [19, 21, 24, 34, 35, 37, 43, 45, 46]. Strzelcyk et al. found Beck depression inventory scores were significantly higher among DS caregivers, when compared to caregivers of children with DRE and seizure remission groups [46]. Three papers reported a general negative impact on emotional well-being or mental exhaustion and 15% of caregivers requested additional emotional counseling [30, 39, 41]. Caregivers take more psychotropic medications than the general public, Rentz et al., found 19.3% of caregivers were taking psychotropic or antidepressant medications, which is nearly twice the national average (11%) [43].

Kopp et al. found 46.5% of parents earned clinically significant scores (T score ⩾ 65) on the Parenting Stress Index [24]. Subscale analysis indicated that elevated total stress scores resulted from various factors, including seizures within the past 6 months, dysfunctional parent-child interactions, difficult child characteristics, a history of psychiatric diagnosis, low intellectual function, and elevated behavioral issues [24]. Selected statements made by caregivers are summarized in Table 4.

Higher rates of depression, anxiety, and stress negatively impact caretaker QOL [43, 45]. Seven studies quantitatively measured QOL among caregivers [21, 24, 29, 30, 43, 45, 46]. All but one found caregivers experienced decreased QOL. Stryzelcyk et al. found similar QOL to that of the average German population; however, they did find higher depression rates among DS caregivers [45]. QOL of DS caregivers was reduced even compared with caregivers of children with drug-resistant epilepsy and seizure-remission groups [46]. Willems et al. found significantly lower QOL among female caregivers compared with males; however, this finding is limited due to a lack of male survey participants [21]. Decreased QOL further impairs parents’ ability to fulfill caregiver responsibility. This impact of caregiving responsibilities on QOL creates a cycle that reinforces the negative impact of caretaker burden, thereby limiting their ability to cope and recover from the economic, psychosocial, and physical consequences. Using EQ-5D general health visual analogue scale (VAS), Campbell et al. found caregivers of patients with VAS < 65 reported a nearly twofold higher impact on mean work productivity (39.1 vs 76.9), and a 1.5-fold greater impact on leisure activities (55.1 vs 82.6) [29]. Rentz et al. found TSC caregivers who spent greater total hours researching the disease, finding doctors, and scheduling appointments had lower physical and mental health related QOL scores (*p* < 0.0025 and *p* < 0.05, respectively) and higher depressive symptoms scores ( < 0.0001) [43]. Domaradszki et al. found overall, 50.7% and 46.6% reported an inability to cope with depression and stress, respectively [30].

### Impact on physical health

Eight papers reported negative physical impacts of caregiving, with three papers showing significantly lower mean physical component summary scores than the general US population [19, 29, 30, 34, 35, 39, 41, 43]. Mothers of children with DS had a worse perception of their own general health compared with fathers (19.5% vs. 15.3%, respectively, rated their own general health as poor or very poor) [40].

Sleep and fatigue were the most studied physical costs of caregiver responsibilities. Six studies evaluated sleep as a consequence of caregiver responsibilities [30, 33, 34, 37–39]. The impact on sleep may be due to increased stress and coping mechanisms for concerns related to sudden unexpected death in epilepsy (SUDEP) or nocturnal seizures. The use of auditory monitoring tools may also play a role.

Jensen et al. found sleep deprivation to be the most discussed burden by all caregivers, primarily driven by fear of their child experiencing a life-threatening seizure or SUDEP during sleep [35]. In a study examining caregiver sleep and stratifying outcomes into sleep disturbances, caregiver fatigue, and sleep-related impairment, Hesdorffer et al. found that the only factor associated with caregiver disturbance, fatigue, and sleep-related impairment was sharing a bed or room [33]. Use of an audio monitor (29%), a seizure alert device (13%), and keeping the door open (15%), were also associated with sleep disturbance and fatigue, but not with sleep-related impairment. This may suggest caregivers find ways to compensate during their waking hours for the impact of sleep disturbance and fatigue. On the other hand, pediatric nocturnal seizures were associated with caregiver sleep disturbance, but not with caregiver fatigue or sleep-related impairment. Overall, results suggest that caregiver’s sleep patterns were most affected by the unpredictable nature and worry of nocturnal seizures and SUDEP [33].

### Opportunity cost of caregiving responsibilities

Table 2 summarizes the opportunity cost of caregiving. Many studies regarding opportunity cost of caregiving responsibilities used semi-structured interviews and open surveys. Selected statements made my caregivers are summarized in Table 4. Caregivers have decreased leisure time, and their social lives and relationships are impacted by caregiving responsibilities. As caregivers utilize their free time for caregiving responsibilities, this may lead to feelings of isolation and a reduction in their ability to cope with the burden of caregiving. Opportunity cost is driven in part by the unpredictable nature of DEE. Seven papers report leisure time given up; Whittington et al. specifically report a total of ~2047 hours annually [29–31, 36, 39, 47] Twelve papers reported caregiving had a negative impact on relationships with three papers finding lower social functioning scores compared to the general population [19, 30, 31, 34–36, 38–40, 42, 43]. Family relationships were the most frequently impacted.

Nabbout et al. found 18.4% of patient families had not taken a vacation in last 12 months, with 85% attributing this to their child having DS and the unpredictability of seizures. Families that were able to go on vacation indicated that their vacation desinations were heavily influenced by proximity to a PICU [40]. Using the Oberst Caregiving Burden Scale (OCBS), Campbell et al. found caregivers primarily spent their time providing transportation, followed by personal care of patients, additional household tasks, communication, symptom observation, coordinating resources, and then medical or nursing treatment [29].

Opportunity cost extends to siblings as well. Gibson et al. reports that 40% of caregivers have no time for their other children [31]. Lagae et al. found 46% of siblings missed leisure opportunities in the past four weeks so that their parents could take care of their sibling with DS. School attendance was not affected [36]. Among 24 families of DS to complete semi-structured interviews, Nolan et al. found both positive and negative effects. The most common positive effect was a caring nature and interest in helping others. The most common negative effect was decreased attention and resentment of the affected sibling [42].

In a cross-sectional survey of siblings of patients with LGS, DS, and other developmental and epileptic encephalopathies (DEE), Bailey et al. found that 18% of siblings aged 13–17 years and 29% of adult siblings felt that they had “lost their childhood.” Older siblings more frequently felt responsible for patients with DEE (63% of adult vs 41% of adolescent siblings) [28]. Bailey et al. found parents reported siblings of DEE patients had lower grades (20%) and demanded more attention (68%). 29% of parents reported altercations with siblings of DEE patients due to caregiving responsibilities [28].

### Indirect costs are a reinforcing cycle

Resources are available for patients. However, caregivers sometimes describe making sacrifices in order to qualify for social services. Additionally, caregivers report gaps: Graffigna et al. reports 50% of caregivers have unmet needs, and Nolan et al. reports 70% had difficulty finding an alternate caregiver [32, 42]. Fear of seizures and communication barriers, such as issues with expressive communication or receptive communication, with patients make it particularly difficult for parents to defer caregiving responsibilities to paid services [38, 42].

### Positive impact of caregiving responsibilities

Caregiving also has positive impacts, primarily through a sense of fulfillment and personal growth, with up to 90% of caregivers reporting some sense of fulfillment [30, 41]. 74% of parents responded that the experience of having a child with epilepsy brought them and their partner closer together. Themes of self-empowerment, resilience, and personal growth emerged when caregivers explained how having a child with epilepsy affected their families [31].

## Discussion

Caregiver responsibilities of developmental epileptic encephalopathies occupy a considerable amount of time. Responsibilities cannot be easily supplemented or transferred to paid services due to the complex nature of the disease and patients’ communication limitations. Caregiving responsibilities place a substantial economic burden on families, primarily measured through productivity loss and changes in employment status [4, 8, 48]. This indirect cost disproportionately impacts female caregivers [20, 46]. Families report rearranging their lives—relocating, forgoing vacations, and sacrificing leisure time [29, 30, 38, 40, 47]. Feelings of loss of control, stress from the unknown, and worry about the future of their child are described [24, 32, 35, 38, 39]. Decreased quality of life and increased levels of anxiety, depression, and stress are seen among caregivers compared to peers in the population [19, 35, 37, 43]. Caregiving takes a toll on physical well-being leading to pain, fatigue, and sleep-related impairments [29, 30, 35, 37–41]. This leads to a decline in the physical health of caregivers compared to the general public [19, 35, 43]. Decreased leisure time leads parents to sacrifice their hobbies, personal ambitions, and interests, and has a negative impact on their social lives and relationships. Consequently, feelings of isolation are reported, which in turn limits their ability to effectively cope with the overall burden of caregiving [29, 30, 35, 38, 42, 43, 47].

Caregiving has positive aspects such as a sense of fulfillment, stronger family relationships, resilience, and personal growth [30, 41]. The overall measured impact of caregiving responsibilities tends to be reported with economic, psychosocial, and physical ramifications on caretakers well-being. Our findings suggest that this impact extends beyond parents and may also involve siblings, although the literature on siblings is generally lacking [28, 36, 42]. Patient characteristics such as communication impairment, behavioral issues, sleep issues, and frequent seizures correlate with higher caregiver burden and indirect costs [21, 33]. For this reason, it is more difficulty for parents to defer caregiving responsibilities to paid services, even if resources are not limited.

In the United States, there are programs to relieve indirect costs incurred by caregivers of children with developmental epileptic encephalopathies. These include Supplemental Security Income (SSI), the only source of federal income support targeted to families caring for children with disabilities. To qualify for SSI, total pre-tax monthly income is limited to $1,913 for one individual and $2,827 for a couple and countable assets are limited to $2000 if the child lives with one parent, or $3000 if the child lives with two parents. SSI benefits average $650/month for a child with disability [49]. The Family and Medical Leave Act (FMLA) allows eligible employees to take unpaid leave to care for a family member with serious health condition, including children, without immediate threat of dismissal from the position. The maximum allowable protected length of time is 12 weeks per year [50]. There are no prescriptions for how employers account for loss of these employees’ work time [51]. Some disease specific patient advocacy organizations provide communities to help with families cope with caregiver burden. Many of these are typically from philanthropic support for one-time gifts. For example, the caregiver foundation of a non-profit based in Hawaii provides caregiver workshops, helps coordinate services and provides financial planning [52]. With this study we provide a summary of the existing literature and a framework for future investigation. For example, it may not be straightforward to qualify for social services, and caregivers may find that the services provided are inadequate in terms of both quantity and quality [35, 49]. Overrall, 50% of caregivers reported unmet needs, while 70% had difficulty finding an alternate caregiver [32, 42]. The indirect costs combined with barriers to access of resources create a reinforcing cycle, compounding the difficulties experienced by caregivers.

Strzelczyk et al. provide a comprehensive systematic review of the burden of DS, offering a detailed quantitative analysis that includes epidemiology, mortality, health-related QOL, and a strong focus on direct economic costs. The study also highlights the profound impact on caregivers, particularly in terms of quality-of-life disruptions and productivity losses. In contrast, this systematic review takes a broader approach by examining multiple DEE, including DS, TSC and LGS. This review highlights both shared indirect costs across syndromes and those unique to each condition. Rather than focusing on epidemiology and direct healthcare costs, it primarily explores indirect costs and their psychosocial effects on families. Through the discussion of themes such as caregiver career sacrifices, emotional strain, social isolation, and even some positive aspects of caregiving, such as personal growth, this review provides a broader perspective on the overall caregiver experience.

Overall, there is a paucity of research regarding the indirect cost to caregivers of patients LGS, TSC, and DS. To our knowledge, there has been no study published regarding the indirect costs of caregiving responsibilities for LGS patients beyond interview-based qualitative studies. The studies included in this review carry a high risk of bias, primarily due to limitations in study design and small sample sizes. While these factors may somewhat limit the generalizability of the findings, they do not diminish the importance of the insights gained. The high risk of bias also suggests that the current evidence base may not fully capture the complexity or variability of indirect costs in patients with DEE. This further highlights a significant gap in the literature and underscores the need for more rigorous, large-scale studies to better understand and quantify the indirect costs for patients with DEE. In order to bridge this gap, semi-structured interviews can be paired with quantitative surveys such as the Work Productivity and Activity Impairment Questionnaire and the Impact on Family Scale. This would provide the ability to catalog the indirect costs and burden of care specifically amongst LGS caregivers and estimate associated the monetary costs.

Limitations include a high risk of bias due to the study design and lack of comparison group for many of the studies. No studies were found investigating the impact or efficacy of policies or initiatives intended to alleviate indirect costs and caregiver burden. Few studies included demographic data detailing socioeconomic status and race or ethnicity. Socioeconomic factors may constitute a key component of adherence in pediatric epilepsy [5]. Studies that did include demographic and socioeconomic data showed subjects were primarily female, highly educated, and Non-Hispanic White. Understanding caregiver burden among patients from a diverse and inclusive perspective is essential. Furthermore, this systematic review is also limited to studies published prior to May 2023 and to the three databases searched which is not exhaustive.

## Conclusions

The indirect cost and caregiver burden to families of patients with developmental epileptic encephalopathies are significant, and contributors are multifactorial. Economic, psychosocial, and physical aspects on caregivers impacts not only parents but also the entire family unit, including siblings. Further studies are necessary to understand and quantify the total indirect cost and caregiver burden for families of patients with DEE. It is essential to develop this knowledge for families to make informed health care decisions to facilitate sustainable long-term outcomes. Subsequently, directions for the future include health care delivery design and implementation of interventions and policies to address and alleviate these indirect costs and caregiver burden.

## Electronic supplementary material

Below is the link to the electronic supplementary material.

Supplementary Material 1

Supplementary Material 2

## Data Availability

All data relevant to the study are included in the article or uploaded as supplementary information.

## Abbreviations

DEE: Developmental and Epileptic Encephalopathies
DRE: Drug-resistant Epilepsy
LGSL: Lennox-Gastaut Syndrome
DS: Dravet Syndrome
TSC: Tuberous Sclerosis Complex
BDI-II: Beck Depression Inventory
PRISMA: Preferred Reporting Items for Systematic Reviews and Meta-Analyses
QOL: Quality of Life
EQ-5D: EuroQol-ED
VAS: Visual Analogue Scale
SUDEP: Sudden Unexpected Death in Epilepsy
OCBS: Oberst Caregiving Burden Scale
FMLA: Family and Medical Leave Act

## References

[1] Boccuzzi SJ. Indirect health care costs. In: Weintraub W, editor. Cardiovascular health care economics. Totowa, NJ: Humana Press; 2003. p. 63–79.

[2] Sabermahani A, Sirizi MJ, Zolala F, Nazari S. Out-of-pocket costs and importance of nonmedical and indirect costs of inpatients. Value Health Reg Issues. 2021;24:141–47. 33578362 10.1016/j.vhri.2020.05.004

[3] Weintraub WS. The economic burden of illness. JAMA Netw Open. 2023;6(3):e232663. 36912842 10.1001/jamanetworkopen.2023.2663

[4] Bauer JM, Sousa-Poza A. Impacts of informal caregiving on caregiver employment, health, and family. J Popul Ageing. 2015;8(3):113–45.

[5] Huber R, Weber P. Is there a relationship between socioeconomic factors and prevalence, adherence and outcome in childhood epilepsy? A systematic scoping review. Eur J Paediatr Neurol. 2022;38:1–6. 35248913 10.1016/j.ejpn.2022.01.021

[6] Beghi E, Frigeni B, Beghi M, De Compadri P, Garattini L. A review of the costs of managing childhood epilepsy. Pharmacoeconomics. 2005;23(1):27–45. 15693726 10.2165/00019053-200523010-00003

[7] Gao L, Xia L, Pan SQ, Xiong T, Li SC. Burden of epilepsy: a prevalence-based cost of illness study of direct, indirect and intangible costs for epilepsy. Epilepsy Res. 2015;110:146–56. 25616467 10.1016/j.eplepsyres.2014.12.001

[8] Strzelczyk A, Reese JP, Dodel R, Hamer HM. Cost of epilepsy: a systematic review. Pharmacoeconomics. 2008;26(6):463–76. 18489198 10.2165/00019053-200826060-00002

[9] Russ SA, Larson K, Halfon N. A national profile of childhood epilepsy and seizure disorder. Pediatrics. 2012;129(2):256–64. 22271699 10.1542/peds.2010-1371

[10] Begley C, Wagner RG, Abraham A, Beghi E, Newton C, Kwon CS, et al. The global cost of epilepsy: a systematic review and extrapolation. Epilepsia. 2022;63(4):892–903. 35195894 10.1111/epi.17165

[11] Jakobsen AV, Moller RS, Nikanorova M, Elklit A. The impact of severe pediatric epilepsy on experienced stress and psychopathology in parents. Epilepsy Behav. 2020;113:107538. 33238238 10.1016/j.yebeh.2020.107538

[12] Baca CB, Vickrey BG, Hays RD, Vassar SD, Berg AT. Differences in child versus parent reports of the child’s health-related quality of life in children with epilepsy and healthy siblings. (1524-4733 (Electronic)). 10.1111/j.1524-4733.2010.00732.x PMC3065295 20561342

[13] Yu Z, Shao Q, Hou K, Wang Y, Sun X. The experiences of caregivers of children with epilepsy: a meta-synthesis of qualitative research studies. (1664-0640 (Print)). 10.3389/fpsyt.2022.987892 PMC9513543 36177220

[14] Scheffer IE, Berkovic S, Capovilla G, Connolly MB, French J, Guilhoto L, et al. ILAE classification of the epilepsies: position paper of the ILAE commission for classification and terminology. Epilepsia. 2017;58(4):512–21. 28276062 10.1111/epi.13709 PMC5386840

[15] Raga S, Specchio N, Rheims S, Wilmshurst JM. Developmental and epileptic encephalopathies: recognition and approaches to care. Epileptic Disord. 2021;23(1):40–52. 33632673 10.1684/epd.2021.1244

[16] Scheffer IE, Liao J. Deciphering the concepts behind Epileptic encephalopathy and Developmental and epileptic encephalopathy. Eur J Paediatr Neurol. 2020;24:11–14. 31926847 10.1016/j.ejpn.2019.12.023

[17] Specchio N, Curatolo P. Developmental and epileptic encephalopathies: what we do and do not know. Brain. 2021;144(1):32–43. 33279965 10.1093/brain/awaa371

[18] Yousaf MN, Naqvi HA, Kane S, Chaudhary FS, Hawksworth J, Nayar VV, et al. Cerebrospinal fluid liver pseudocyst: a bizarre long-term complication of ventriculoperitoneal shunt: a case report. World J Hepatol. 2023;15(5):715–24. 10.4254/wjh.v15.i5.715 PMC10251282 37305372

[19] Gallop K, Wild D, Verdian L, Kerr M, Jacoby A, Baker G, et al. Lennox-Gastaut Syndrome (LGS): development of conceptual models of health-related quality of life (HRQL) for caregivers and children. Seizure. 2010;19(1):23–30. 19948417 10.1016/j.seizure.2009.10.007

[20] Grau J, Zöllner JP, Schubert-Bast S, Kurlemann G, Hertzberg C, Wiemer-Kruel A, et al. Direct and indirect costs and cost-driving factors of Tuberous sclerosis complex in children, adolescents, and caregivers: a multicenter cohort study. Orphanet J Rare Dis. 2021;16(1):282. 34154622 10.1186/s13023-021-01899-x PMC8218507

[21] Willems LM, Schubert-Bast S, Grau J, Hertzberg C, Kurlemann G, Wiemer-Kruel A, et al. Health-related quality of life in children and adolescents with tuberous sclerosis complex and their caregivers: a multicentre cohort study from Germany. Eur J Paediatr Neurol. 2021;35:111–22. 34673401 10.1016/j.ejpn.2021.10.003

[22] Strzelczyk A, Lagae L, Wilmshurst JM, Brunklaus A, Striano P, Rosenow F, et al. Dravet syndrome: a systematic literature review of the illness burden. Epilepsia Open. 2023;8(4):1256–70. 37750463 10.1002/epi4.12832 PMC10690674

[23] Sullivan J, Deighton AM, Vila MC, Szabo SM, Maru B, Gofshteyn JS, et al. The clinical, economic, and humanistic burden of Dravet syndrome - a systematic literature review. Epilepsy Behav. 2022;130:108661. 35334258 10.1016/j.yebeh.2022.108661

[24] Kopp CM, Muzykewicz DA, Staley BA, Thiele EA, Pulsifer MB. Behavior problems in children with tuberous sclerosis complex and parental stress. Epilepsy Behav. 2008;13(3):505–10. 18602868 10.1016/j.yebeh.2008.05.010

[25] Page MJ, McKenzie JE, Bossuyt PM, Boutron I, Hoffmann TC, Mulrow CD, et al. The PRISMA, 2020 statement: an updated guideline for reporting systematic reviews. BMJ. 2021;372. 10.1136/bmj.n71 PMC8005924 33782057

[26] Shadish WR, Cook TD, Campbell DT. Experimental and quasi-experimental designs for generalized causal inference. Houghton: Mifflin and Company; 2002.

[27] Sterne JA, Hernán MA, Reeves BC, Savović J, Berkman ND, Viswanathan M, et al. ROBINS-I: a tool for assessing risk of bias in non-randomised studies of interventions. BMJ. 2016;355(i4919). 10.1136/bmj.i4919 PMC5062054 27733354

[28] Bailey LD, Schwartz L, Dixon-Salazar T, Meskis MA, Galer BS, Gammaitoni AR, et al. Psychosocial impact on siblings of patients with developmental and epileptic encephalopathies. Epilepsy Behav. 2020;112:107377. 32846306 10.1016/j.yebeh.2020.107377

[29] Campbell JD, Whittington MD, Kim CH, VanderVeen GR, Knupp KG, Gammaitoni A. Assessing the impact of caring for a child with Dravet syndrome: results of a caregiver survey. Epilepsy Behav. 2018;80:152–56. 29414545 10.1016/j.yebeh.2018.01.003

[30] Domaradzki J, Walkowiak D. Emotional experiences of family caregivers of children with Dravet syndrome. Epilepsy Behav. 2023;142:109193. 37028149 10.1016/j.yebeh.2023.109193

[31] Gibson PA. Lennox-Gastaut syndrome: impact on the caregivers and families of patients. J Multidiscip Healthc. 2014;7:441–48. 25336963 10.2147/JMDH.S69300 PMC4199842

[32] Graffigna G, Bosio C, Cecchini I. Assisting a child with tuberous sclerosis complex (TSC): a qualitative deep analysis of parents’ experience and caring needs. BMJ Open. 2013;3(12):e003707. 24319280 10.1136/bmjopen-2013-003707 PMC3855572

[33] Hesdorffer DC, Kroner BL, Shen J, Farrell K, Roberds S, Fureman B. Factors associated with caregiver sleep quality related to children with rare epilepsy syndromes. J Educ Chang Pediatrics: X. 2020;2. 10.1016/j.ympdx.2020.100021 PMC10236549 37332626

[34] Jansen AC, Vanclooster S, de Vries PJ, Fladrowski C, Beaure d’Augères G, Carter T, et al. Burden of illness and quality of life in tuberous sclerosis complex: findings from the TOSCA study. Front Neurol. 2020;11:904. 32982929 10.3389/fneur.2020.00904 PMC7485558

[35] Jensen MP, Liljenquist KS, Bocell F, Gammaitoni AR, Aron CR, Galer BS, et al. Life impact of caregiving for severe childhood epilepsy: results of expert panels and caregiver focus groups. Epilepsy Behav. 2017;74:135–43. 28734197 10.1016/j.yebeh.2017.06.012

[36] Lagae L, Irwin J, Gibson E, Battersby A. Caregiver impact and health service use in high and low severity Dravet syndrome: a multinational cohort study. Seizure. 2019;65:72–79. 30616222 10.1016/j.seizure.2018.12.018

[37] Maltseva M, Schubert-Bast S, Zollner JP, Bast T, Mayer T, von Spiczak S, et al. Sleep quality, anxiety, symptoms of depression, and caregiver burden among those caring for patients with Dravet syndrome: a prospective multicenter study in Germany. Orphanet J Rare Dis. 2023;18(1):98. 37120555 10.1186/s13023-023-02697-3 PMC10148440

[38] Nabbout R, Auvin S, Chiron C, Irwin J, Mistry A, Bonner N, et al. Development and content validation of a preliminary core set of patient- and caregiver-relevant outcomes for inclusion in a potential composite endpoint for Dravet syndrome. Epilepsy Behav. 2018;78:232–42. 29108913 10.1016/j.yebeh.2017.08.029

[39] Nabbout R, Auvin S, Chiron C, Thiele E, Cross H, Scheffer IE, et al. Perception of impact of Dravet syndrome on children and caregivers in multiple countries: looking beyond seizures. Dev Med Child Neurol. 2019;61(10):1229–36. 30828793 10.1111/dmcn.14186

[40] Nabbout R, Dirani M, Teng T, Bianic F, Martin M, Holland R, et al. Impact of childhood Dravet syndrome on care givers of patients with DS, a major impact on mothers. Epilepsy Behav. 2020;108:107094. 32375095 10.1016/j.yebeh.2020.107094

[41] Gil-Nagel A, Sánchez-Carpintero R, Villanueva V. Patient profile, management, and quality of life associated with Dravet syndrome: a cross-sectional, multicentre study of 80 patients in Spain. Sci Rep. 2023;13(1):3355. 36849632 10.1038/s41598-023-30273-z PMC9971205

[42] Nolan KJ, Camfield CS, Camfield PR. Coping with Dravet syndrome: parental experiences with a catastrophic epilepsy. Dev Med Child Neurol. 2006;48(9):761–65. 16904024 10.1017/S0012162206001629

[43] Rentz AM, Skalicky AM, Pashos CL, Liu Z, Magestro M, Pelletier CL, et al. Caring for children with Tuberous sclerosis complex: what is the physical and mental health impact on caregivers? J Child Neurol. 2015;30(12):1574–81. 25838447 10.1177/0883073815575364

[44] Skalicky AM, Rentz AM, Liu Z, Said Q, Nakagawa JA, Frost MD, et al. Economic burden, work, and school productivity in individuals with tuberous sclerosis and their families. J Med Econ. 2018;21(10):953–59. 29890870 10.1080/13696998.2018.1487447

[45] Strzelczyk A, Kalski M, Bast T, Wiemer-Kruel A, Bettendorf U, Kay L, et al. Burden-of-illness and cost-driving factors in Dravet syndrome patients and carers: a prospective, multicenter study from Germany. Eur J Paediatr Neurol. 2019;23(3):392–403. 30871879 10.1016/j.ejpn.2019.02.014

[46] Strzelczyk A, Schubert-Bast S, Bast T, Bettendorf U, Fiedler B, Hamer HM, et al. A multicenter, matched case-control analysis comparing burden-of-illness in Dravet syndrome to refractory epilepsy and seizure remission in patients and caregivers in Germany. Epilepsia. 2019;60(8):1697–710. 31247127 10.1111/epi.16099

[47] Whittington MD, Knupp KG, Vanderveen G, Kim C, Gammaitoni A, Campbell JD. The direct and indirect costs of Dravet syndrome. Epilepsy Behav. 2018;80:109–13. 29414539 10.1016/j.yebeh.2017.12.034

[48] White D, DeAntonio D, Ryan B, Colyar M. The economic impact of caregiving BlueCross BlueShield. 2021, November, 8;2021.

[49] Romig K. SSI: a lifeline for children with disabilities. Cent Budget Policy Priorities. 2017, May, 11;2017.

[50] Labor USDo. Family and medical leave act.

[51] Mitchell RJ, Bates P. Measuring health-related productivity loss. Popul Health Manag. 2011;14(2):93–98. 21091370 10.1089/pop.2010.0014 PMC3128441

[52] Foundation TC. The caregiver foundation-services. Available from: https://thecaregiverfoundation.org/the-caregiver-foundation-services.

