# Supplementary material for: Systematic review of indirect costs to families of children with developmental epileptic encephalopathies
Source: Orphanet J Rare Dis. 2025 Nov 12;20:579. doi: 10.1186/s13023-025-04081-9 (PMC12613530; doi:10.1186/s13023-025-04081-9)
Supplement: Supplementary file 1 — Supplementary Material 1 [file 13023_2025_4081_MOESM1_ESM.docx]

| **Supplementary Table 1.** Search Terms | |
| --- | --- |
| **Database** | **Terms** |
| **PubMed**  **1,106** | #1 AND #2 AND #3    (((((Dravet*) OR ('severe myoclonic epilepsy') OR (Tuberous Sclerosis*) OR (TSC[Title/Abstract])) OR (Tuberous Sclerosis complex*)) OR (Lennox-gastaut*)) OR (Lennox Gastaut*)OR (LGS[Title/Abstract]) OR (Lennox Syndrome*))    ((((((((((((((((((((cost*) OR ('burden of illness')) OR ('cost-benefit analysis'[MeSH Terms])) OR ('cost of illness')) OR ('health care costs*)) OR ('indirect cost*')) OR ('productivity cost*')) OR ('human capital')) OR ('quality of life')) OR ('sickness impact profile')) OR ('disability rate*)) OR ('burden cost*')) OR ('productivity loss')) OR (caregiver)) OR (parent*)) OR (parental)) OR (guardian)) OR (career))OR ('cost* of care')) OR ('cost and cost analysis'[MeSH Terms])    ((((((((child[MeSH Terms]) OR (child*)) OR (adolescent[MeSH Terms])) OR (adolescent*)) OR (teen*)) OR (youth*)) OR (infant*)) OR (pediatric*)) OR (pediatric[MeSH Terms]) |
| **Embase**  **14** | #1 AND #2 AND #3    ((Dravet*)) OR (('severe myoclonic epilepsy*')) OR (('tuberous sclerosis*')) OR ((TSC):ti,ab,kw) OR (('tuberous sclerosis complex')) OR ((Lennox-Gastaut*)) OR (('Lennox Gastaut*')) OR ((LGS):ti,ab,kw) OR (('lennox syndrome*'))    (child)/br OR ((child)/exp/mj) OR (adolescent*) OR ((adolescent)/exp/mj) OR (teen*) OR (youth*) OR (infant*) OR (pediatric*) AND ((pediatrics)/exp/mj)    cost* OR 'cost benefit analysis'/exp/mj OR 'cost benefit analysis' OR 'burden of illness'/exp OR 'burden of illness' OR 'health care costs*' OR 'indirect cost*' OR 'productivity cost*' OR 'human capital'/exp OR 'human capital' OR 'quality of life'/exp OR 'quality of life' OR 'cost of illness'/exp OR 'cost of illness' OR 'sickness impact profile'/exp OR 'sickness impact profile' OR 'disability rate' OR 'burden cost*' OR 'productivity loss'/exp OR 'productivity loss' OR 'caregiver'/exp OR 'caregiver' OR 'parent'/exp OR 'parent' OR 'parental' OR 'guardian'/exp OR 'guardian' OR 'career'/exp OR 'career' OR 'cost of care |
| **Scopus**  **964** | ( ( TITLE-ABS-KEY ( dravet* ) OR TITLE-ABS-KEY ( 'severe AND myoclonic AND epilepsy' ) OR TITLE-ABS-KEY ( tuberous AND sclerosis* ) OR TITLE-ABS-KEY ( tuberous AND sclerosis AND complex* ) OR TITLE-ABS-KEY ( lennox-gastaut* ) OR TITLE-ABS-KEY ( lennox AND gastaut* ) OR TITLE-ABS-KEY ( lgs ) OR TITLE-ABS-KEY ( lennox AND syndrome* ) ) ) AND ( ( TITLE-ABS-KEY ( child* ) OR TITLE-ABS-KEY ( adolescent* ) OR TITLE-ABS-KEY ( teen* ) OR TITLE-ABS-KEY ( youth* ) OR TITLE-ABS-KEY ( infant* ) OR TITLE-ABS-KEY ( pediatric* ) ) ) AND ( ( TITLE-ABS-KEY ( cost* ) OR TITLE-ABS-KEY ( 'burden AND of AND illness' ) OR TITLE-ABS-KEY ( 'cost-benefit AND analysis' ) OR TITLE-ABS-KEY ( 'cost AND of AND illness' ) OR TITLE-ABS-KEY ( 'health AND care AND costs*' ) OR TITLE-ABS-KEY ( 'indirect AND cost' ) OR TITLE-ABS-KEY ( 'productivity AND cost' ) OR TITLE-ABS-KEY ( 'human AND capital' ) OR TITLE-ABS-KEY ( 'quality AND of AND life' ) OR TITLE-ABS-KEY ( 'sickness AND impact AND profile' ) OR TITLE-ABS-KEY ( 'disability AND rate*' ) OR TITLE-ABS-KEY ( 'burden AND cost*' ) OR TITLE-ABS-KEY ( 'productivity AND loss' ) OR TITLE-ABS-KEY ( 'caregiver' ) OR TITLE-ABS-KEY ( 'parent*' ) OR TITLE-ABS-KEY ( 'parental' ) OR TITLE-ABS-KEY ( guardian ) OR TITLE-ABS-KEY ( career ) OR TITLE-ABS-KEY ( 'cost* AND of AND care' ) OR TITLE-ABS-KEY ( 'cost AND cost AND analysis' ) ) ) |
