# Supplementary material for: Systematic review of indirect costs to families of children with developmental epileptic encephalopathies
Source: Orphanet J Rare Dis. 2025 Nov 12;20:579. doi: 10.1186/s13023-025-04081-9 (PMC12613530; doi:10.1186/s13023-025-04081-9)
Supplement: Supplementary file 2 — Supplementary Material 2 [file 13023_2025_4081_MOESM2_ESM.docx]

| Supplementary Table 2. Inclusion and Exclusion Criteria | | |
| --- | --- | --- |
| Characteristics | Inclusion criteria | Exclusion criteria |
| Language | English or translated to English | Non-English |
| Time Period | From inception of database to 2023 |  |
| Study Design | RCTs, non-RCTS; controlled before-&-after studies; interrupted rime series studies; noncontrolled before-&-after studies; cohort studies; cross-sectional studies; semi-structured interviews; focus groups | Systematic reviews, conference abstracts, case reports, letters, comments, editorials and review papers |
| Context | Pediatric patient population with Lennox-Gastaut, Dravet Syndrome, Tuberous Sclerosis or another childhood epilepsy syndrome |  |
| Outcomes | Studies that consider indirect costs to caregivers and families:   - Economic cost - Psychosocial impact - Physical impact - Impact on siblings | Studies that did not address cost in their result section or only report direct costs |

RCT, randomized control trial
